# Study protocol for the evaluation of *Fear-Less*: a stepped-care program for fear of cancer recurrence in survivors with early-stage disease

**DOI:** 10.1186/s40814-022-01123-y

**Published:** 2022-08-10

**Authors:** Mei Jun Tran, Michael Jefford, Ben Smith, Fiona Lynch, Haryana M. Dhillon, Joanne Shaw, Lachlan McDowell, Alan White, Clare Halloran, David Wiesenfeld, Maria Ftanou

**Affiliations:** 1 Psychosocial Oncology Program, Peter MacCallum Cancer Centre, Melbourne, Victoria Australia; 2 Australian Cancer Survivorship Centre, Peter MacCallum Cancer Centre, Melbourne, Victoria Australia; 3 Department of Health Services Research, Peter MacCallum Cancer Centre, Melbourne, Victoria Australia; 4 Sir Peter MacCallum Department of Oncology, University of Melbourne, Melbourne, Victoria Australia; 5 Ingham Institute for Applied Medical Research and South West Sydney Clinical Campuses, University of New South Wales, Sydney, NSW Australia; 6 Andrew Love Cancer Centre, Barwon Health, Geelong, Victoria Australia; 7 Faculty of Science, School of Psychology, Psycho-Oncology Cooperative Research Group, The University of Sydney, Camperdown, NSW Australia; 8 Faculty of Science, School of Psychology, Centre for Medical Psychology & Evidence-based Decision-making, The University of Sydney, Camperdown, NSW Australia; 9 Department of Radiation Oncology, Peter MacCallum Cancer Centre, Melbourne, Victoria Australia; 10 Consumer Representative, Peter MacCallum Cancer Centre, Melbourne, Victoria Australia; 11 Consumer Representative, Continence Foundation of Australia, Melbourne, Victoria, Australia; 12 Director Head and Neck Tumour Stream, Melbourne Health and Peter MacCallum Cancer Centre, Melbourne, Victoria Australia; 13 Lead in Research and Education Head and Neck, VCCC Alliance, Melbourne, Victoria Australia; 14 Honorary Clinical Professor, The University of Melbourne, Melbourne, Victoria Australia; 15 Melbourne School of Population and Global Health, The University of Melbourne, Melbourne, Victoria Australia

**Keywords:** Fear of cancer recurrence, Cancer survivorship, Stepped-care, Psychology

## Abstract

**Background:**

Fear of cancer recurrence (FCR) is a significant unmet need amongst cancer survivors and is consistently associated with psychological distress and impaired quality of life. Psychological interventions for FCR, such as ConquerFear, have demonstrated efficacy in reducing FCR and improving emotional wellbeing. Unfortunately, there are barriers to the uptake of evidence-based FCR treatments in clinical practice. A stepped-care FCR treatment model may overcome these barriers and has demonstrated potential in people with advanced melanoma. This study aims to evaluate the acceptability, feasibility, and impact of a stepped-care FCR treatment model (*Fear-Less*) in people with other cancer types, who have completed treatment with curative intent.

**Methods:**

Sixty people with early-stage cancer (defined as individuals who have received treatment with curative intent and with no metastatic disease) will be screened for FCR using the Fear of Cancer Recurrence Inventory—Short Form (FCRI-SF). Individuals reporting moderate FCR (FCRI-SF between 13 and 21) will be offered a clinician-guided self-management resource; those reporting high FCR (FCRI-SF ≥ 22) will be offered individual therapy according to the ConquerFear protocol. Participants will complete purpose-built evaluation surveys assessing their FCR screening and intervention experiences. Clinicians will also complete a survey regarding their experiences of the treatment model. *Fear-Less* will be evaluated in terms of (1) acceptability (i.e., patient and clinician experience), (2) feasibility (i.e., referral uptake, treatment adherence, and time taken to screen and deliver interventions), and (3) impact (i.e., pre- to post-intervention FCR changes).

**Discussion:**

The *Fear-Less* stepped-care model is a novel framework for screening FCR and stratifying survivors to the appropriate level of treatment. Our study will provide an indication of whether *Fear-Less* is a feasible and acceptable FCR model of care amongst survivors with early-stage disease and inform further investigations of this model.

**Trial registration:**

Australian New Zealand Clinical Trials Registry (ANZCTR); ACTRN12622000818730.

**Supplementary Information:**

The online version contains supplementary material available at 10.1186/s40814-022-01123-y.

## Background

Fear of cancer recurrence (FCR) describes the fear, worry, or concern cancer will come back or progress [1]. Approximately, 50% of cancer survivors experience moderate-to-high or clinically significant FCR [2, 3]. FCR involves intrusive thoughts, worries, hypervigilance to physical symptoms and either avoidance of, or excessive levels of symptom checking [1]. At clinical levels, FCR is characterized by high levels of persistent worry and hypervigilance or hypersensitivity to bodily symptoms [4] and has been associated with poor day-to-day functioning, reduced quality of life, and increased rates of depression, anxiety, and worry about the future [5]. It is often heightened prior to receiving scan results or attending medical reviews [6]. Untreated FCR has been related to the avoidance of or excessive requests for medical reviews, examinations, or follow-ups, potentially risking adverse health outcomes for survivors and increased health costs to survivors and health services [5, 7–9].

Over the past decade, several psychological interventions have been developed and trialled to address cancer survivors’ FCR [7, 10–17]. These interventions target attitudes, beliefs, and behaviors that may exacerbate or maintain FCR. In addition to significantly reducing levels of FCR, these interventions have led to improvement in emotional wellbeing [10, 11] and quality of life [10]. One intervention is ConquerFear, a five-session manualized treatment [10] developed based on a cognitive processing model of FCR [18]. The intervention targets key processes that contribute to FCR, including worry and excessive threat monitoring, unhelpful beliefs about worry, inappropriate monitoring and screening behaviors, and existential concerns [10]. Compared to a manualized relaxation intervention, ConquerFear was shown to reduce FCR in survivors with early-stage melanoma, breast cancer, or colorectal cancer, with reductions maintained at 6 months. Effects of ConquerFear were shown to be mediated by changes in metacognitions and intrusive thoughts about cancer [19]. ConquerFear was also considered by clinicians to be a sustainable intervention in routine practice [20] and likely cost-effective [21].

Unfortunately, support for managing FCR has been rated the top unmet need for cancer survivors in Australia [22], where barriers remain to the uptake of evidence-based FCR treatment in clinical practice. These barriers include lack of routine screening for FCR (e.g., due to the length of screening measures) [22, 23] and the limited availability of therapists with specialized training to deliver FCR interventions such as ConquerFear [24]. Specific strategies are required to address the systemic barriers to delivery of FCR interventions. The use of the limited available resources is also critical to successful implementation.

A stepped-care approach may provide a solution to these barriers. This approach involves offering treatments of differing intensity tailored to survivors’ level of symptoms that can be identified through routine screening. For mild-to-moderate symptoms, a low-intensity intervention is offered that is accessible and convenient for survivors and requires minimal specialist time to deliver [25, 26]. Examples include brief therapy or a self-help approach. In contrast, a high-intensity intervention, such as traditional individual therapy, is offered for people reporting high levels of symptoms. This approach may be more cost-effective compared to the usual care [27] and may increase appropriate referrals to psychosocial services among cancer patients [28]. Stepped-care models have demonstrated benefits for cancer survivors in the management of insomnia [29] and psychological distress [30].

Similar benefits may accrue for FCR, where a stepped-care approach provides a framework to support FCR screening and appropriate referrals to treatment. This approach has been recommended by Cancer Australia (Australia’s peak national government cancer agency) to manage FCR [31] and could improve accessibility to FCR treatment, given most oncology services have limited clinical resources to support all survivors with heightened FCR [24, 26]. In this model, the more intensive intervention (e.g., ConquerFear) is reserved for people with clinical FCR. This is in keeping with findings that ConquerFear appears more efficacious amongst survivors treated for early-stage cancer who presented with higher baseline FCR scores [19]. Lower intensity interventions can then be offered to survivors experiencing mild-to-moderate levels of FCR. Currently, there is no consensus regarding the type of low-intensity intervention that would be best suited to treat FCR [26]. One option is a guided self-management, which has shown promise for improving FCR. For example, an intervention consisting of a psychoeducational resource accompanied by three 15-min telephone sessions was found to improve FCR among early-stage melanoma survivors [11].

On this basis, our team developed the *Fear-Less* stepped-care model for treating FCR. The lower-intensity step consists of a clinician-guided self-management intervention, which is a purpose-developed resource providing education about FCR and cognitive behavioral strategies to manage FCR. The high-intensity step consists of individual therapy using ConquerFear. This stepped-care model was previously evaluated among survivors with stage IV melanoma treated with immunotherapy and targeted therapies [32]. In this study, survivors were screened for FCR and stratified to the above interventions based on their screening scores. The findings indicated *Fear-Less* was acceptable and feasible to implement for both survivors and clinicians. However, this model has yet to be investigated among early-stage cancer survivors.

### Study aims

Our study aims to extend the *Fear-Less* stepped-care model to people with other types of cancer with early-stage disease. For our study, we have defined “early-stage disease” as survivors who have been treated with curative intent and who do not have metastatic disease. As per the *Fear-Less* model of care, individuals with moderate levels of FCR will be offered a clinician-guided self-management intervention and those with clinical levels of FCR will be offered ConquerFear.

The primary aim of our study is to evaluate the acceptability and feasibility of *Fear-Less* to treat FCR in survivors with early-stage disease. This will include evaluations from the perspectives of both survivors and clinicians. A preliminary evaluation of the impact of the stepped-care program on survivors’ FCR levels will also be conducted.

A second, exploratory aim is to assess a one-item measure of FCR, given a barrier to routine screening is the length of existing measures.

## Methods

### Study design

This study is a single-arm evaluation study which will assess the feasibility, acceptability, and impact of *Fear-Less*. The study adheres to the SPIRIT checklist (see Additional File 1).

### Evaluation of Fear-Less by cancer survivors

#### Participants

The participants will include people with breast, gynecology, or head and neck cancer who have completed treatment with curative intent and do not have metastatic disease.

##### Sample size

Sixty survivors will be recruited. As there is currently no clear consensus regarding appropriate sample size for feasibility studies [33], our target sample size is based on our previous feasibility study for *Fear-Less* amongst survivors with stage IV melanoma [32] and on available funding, resources (e.g., clinician time to deliver the stepped-care interventions), and study duration of 18 months. This sample size is also in line with previous pilot and feasibility trials [34].

##### Inclusion criteria

Survivors must meet the following inclusion criteria to participate in the study:

- Aged ≥ 18 years
- Have completed treatment with curative intent. Individuals receiving maintenance therapies, such as endocrine therapy, can be included in the study.
- Able to read and write English
- Able to give written informed consent

##### Exclusion criteria

- Survivors diagnosed with metastatic disease

##### Recruitment and consent

Participants will be recruited from the breast, gynecology, and head and neck clinical services at Peter MacCallum Cancer Centre (Peter Mac), Royal Melbourne Hospital, and Royal Women’s Hospital in Melbourne, Australia. Survivors will complete FCR screening measures (described below) as part of their routine care, for example, during an appointment with their oncologist or by nursing staff completing survivorship care.

Survivors who complete screening and meet the above inclusion criteria will be identified by clinical staff supporting the *Fear-Less* program, such as their oncologist or nurse. These survivors will be provided a leaflet informing them about the *Fear-Less* program and that they will be contacted by a member of the research team. These survivors will then be invited to participate in the evaluation of the stepped-care approach by a member of the research team.

If the survivor is interested in participating, they will be provided with verbal and written information about the project, including the Participant Information and Consent Form (PICF). Consent will be obtained either via an online form or paper form returned using a reply-paid envelope.

#### Study measures

##### Demographics and medical history

Identifying information of the survivor’s name and contact details will be obtained from the medical record and/or consent form only to maintain contact with the patient.

After study consent, survivors’ demographic and clinical characteristics will be collected from their medical record and their treating team. Data collected will include the following:

- Age
- Sex
- Cancer type
- Stage of disease
- Treatment received

##### FCR assessments

FCR screening will be completed using two measures below:

- Fear of Cancer Recurrence Inventory – Short Form (FCRI-SF): The FCRI-SF [35] is a 9-item instrument assessing the presence, frequency, intensity, and duration of thoughts associated with FCR. Total scores range from 0 to 36 with higher scores indicating higher FCR. The FCRI-SF has shown strong internal consistency (*α* = 0.95), temporal stability (*r* = 0.89), and construct validity.
- Fear of Cancer Recurrence – One Item Measure (FCR-1): The FCR-1 is a one-item measure assessing the severity of FCR [23, 36]. Survivors are asked to rate their fear of their cancer returning or getting worse on a scale from 0 (no fear) to 10 (worst possible fear). In its initial validation study, the FCR-1 was responsive to changes in FCR over time and showed concurrent, convergent, and discriminant validity [36]. This measure is included to provide further validation and comparison against the FCRI-SF.

##### Survivor experience surveys

Acceptability of the stepped-care program (i.e., whether *Fear-Less* is palatable and satisfactory to survivors [37]) will be assessed via three purpose-designed survivor experience surveys (see Additional File 2). These surveys were adapted from measures developed for our earlier study evaluating *Fear-Less* amongst survivors with stage IV melanoma [32].

The following surveys will be administered to survivors:

- *The Screening Survivor Experience Survey* will be administered to all participants immediately after providing consent. It is a 10-item measure assessing participants’ prior experience of FCR support (e.g., have they been asked about their FCR levels by a clinician) and includes ratings of the degree to which participants agree or disagree that the screening questionnaires are easy to complete, easy to understand, and acceptable. Open response items ask participants about the importance of screening.
- *The Self-Management Survivor Experience Survey* will be administered to participants 5 weeks following their receipt of the self-management intervention. It is a 10-item measure assessing the most and least useful aspects of the intervention for each participant, whether they would recommend the intervention and subjective changes in FCR.
- *The ConquerFear Survivor Experience Survey* will be administered to participants who complete 5 sessions of ConquerFear. It is a 9-item measure assessing the most and least useful aspects of individual therapy for each participant, whether they would recommend individual therapy and subjective changes in FCR.

##### Process data

**Session checklists** The fidelity of the ConquerFear treatment protocol will be assessed through a review of each session by the psychologists administering the treatment. This will be completed using session checklists included in the ConquerFear protocol. The checklist will be completed at the end of each session.

**Referral rates, uptake, and patient adherence** Feasibility (i.e., the extent to which *Fear-Less* can be successfully implemented to treat FCR [37]) will be assessed by collecting the FCR screening rates, referral rates for each intervention, and intervention uptake and adherence. In particular, the data collected will include the number of survivors: screened, referred to each intervention, declining participation (including reasons), completing measures, and the proportion of the intervention completed (either self-reported percentage of the self-management intervention completed or psychologist reported number of ConquerFear sessions completed). This will be collected using a screening log and case-report form (CRF).

For a list of study measures and process data, see Table 1.

**Table 1:** Study measures

Time point		Pre-enrolment	At enrolment	Post-intervention
**Screening**	FCRI-SF	x		x
	FCR1	x		x
**Survivor experiences**	Screening Survivor Experiences Survey		x	
	Self-Management Survivor Experiences Survey			x
	ConquerFear Survivor Experiences Survey			x
**Process data**	Number of survivors screened	x		
	Number of survivors who declined screening	x		
	Number of survivors referred to each intervention	x		
	Number of participants that consented to the intervention		x	
	Number of survivors declining participation	x		
	Proportion of intervention completed			x
	Number of survivors who completed screening post-intervention			x
	Number of survivors who completed the evaluation			x

#### Study interventions

##### Fear-Less self-management intervention

The self-management intervention is a purpose-designed booklet adapted from a version developed for the evaluation of *Fear-Less* amongst Stage IV melanoma survivors treated with immunotherapies and targeted therapies [32].

The process for adapting the booklet included the following:

1. Editing for applicability to a population treated for early-stage cancer.
2. Review within a focus group with cancer survivors with a range of diagnoses to inform content and design.
3. A review process by the Peter Mac Health Communications Manager and two members of the Peter Mac Consumer Literacy Education and Evaluation Group for feedback regarding the booklet’s design and language.

The feedback provided in steps 2 and 3 informed the development of the final version of the booklet. The self-management booklet includes psychoeducational material about FCR, information about healthy living and wellbeing, and key cognitive behavioral strategies to help address thoughts and behaviors that trigger and maintain FCR. These strategies include identifying triggers for FCR, clarifying values, goal-setting, relaxation, identifying unhelpful thoughts, focusing on things within one’s control, and developing a FCR management plan. As per the initial *Fear-Less* study [32], participants will read the booklet over 5 weeks.

On receipt of the booklet, participants will be provided with education on how to use the booklet, and an opportunity to ask any questions, by a member of the research team. Approximately, 3 weeks after receipt of the self-management booklet, the participant will be contacted by a member of the project team to provide phone support and further education as needed to support participants in using the self-management resource. As per the initial *Fear-Less* study, it is expected the clinician time taken for each of these phone calls will be approximately 20 min [32].

##### ConquerFear

ConquerFear consists of five individual sessions that promote value-based goal setting and teach survivor strategies to manage worry and excessive FCR. Each session will be approximately 60 min every 1–2 weeks and will be facilitated by a psychologist face-to-face or via telehealth. Each session is accompanied by home-based practice of skills learned in session and home reading to consolidate skill acquisition.

In Session 1, a FCR-specific assessment will be conducted and the model of FCR on which the treatment program is based will be introduced [18]. Discussion of existential changes brought about by cancer will be introduced as well as value clarification and goal-setting tasks to be completed for homework.

In Session 2, the impact of vulnerability factors (e.g., past traumatic life events) on the interpretation and meaning of FCR will be discussed. The rationale for the practice of Attention Training Technique (ATT) will be discussed, which aims to help patients reduce their rumination and shift their attention more flexibly when thoughts of recurrence occur. Clients will be asked to practice ATT daily throughout the remainder of the intervention.

In Session 3, Detached Mindfulness will be introduced. This is designed to enhance meta-cognition awareness and the ability to become an objective observer of thought content without evaluating or reacting to cognitions.

In Session 4, information about possible symptom recurrence will be provided and guidelines to help participants distinguish those from benign physical complaints. Self-examination practices and medical surveillance will be reviewed. Avoidant or excessive behaviors will be identified, and a behavioral contract to help clients engage in recommended levels of self-examination and follow-up tests will be developed. Beliefs that may underpin FCR will also be explored through Socratic dialog.

In Session 5, the goal-setting task will be reviewed, as well as skills learned during the intervention. A relapse prevention plan will be developed.

#### Study procedures

Over a 15-week period, eligible survivors who have completed screening will be invited to participate in the *Fear-Less* program. Survivors who provide consent to participate will be stratified into a specific level of care within the stepped-care program based on their screening score on the FCRI-SF [35, 38, 39].

Participants identified as experiencing low FCR (score of <13 on the FCRI-SF) will continue with their usual care with their medical and nursing teams. No further follow-up or additional screening will be undertaken for these participants.

Participants identified as experiencing moderate/subthreshold levels of FCR (score of 13–21 on FCRI-SF) will have a brief discussion with a psychologist on the research team to confirm they are concerned about their level of FCR and would like support for it. Participants who agree to proceed will be offered the clinician-guided self-management intervention.

Participants identified as experiencing high/clinical levels of FCR (score of ≥22 on the FCRI-SF) will have a brief discussion with a psychologist on the research team member to confirm the severity of FCR and whether they would like support for it. Factors that will be assessed during this discussion will include the frequency, emotional impact, functional impact, and avoidance of FCR. Participants whose assessment indicates clinical levels of FCR will be offered ConquerFear. Participants who are considered to have sub-threshold FCR, or who do not wish to take part in the ConquerFear therapy, will be offered the self-management intervention.

Following each intervention, participants will complete the screening questionnaires and a survivor experiences survey relevant to the intervention they received. To minimize missing data, questionnaires and surveys not returned within 2 weeks of receipt will be followed-up by phone and mail/email up to three times, at 2-week intervals. Options for follow-up treatment will also be discussed depending on the survivors’ needs. This may include no further treatment, or referrals to services such as a psychiatrist, additional individual therapy, or peer supports.

**Fig. 1:**
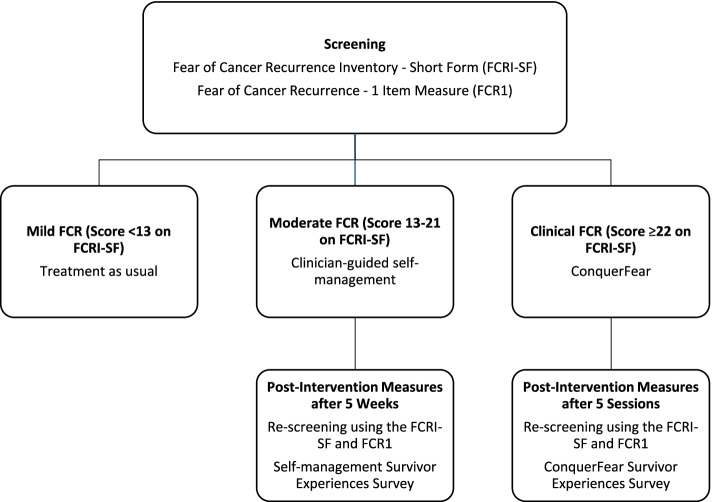
The *Fear-Less* stepped-care model

The three levels of interventions are summarised in Fig. 1.

#### Safety reporting

The potential for adverse events is deemed to be low in this study. In the event that a member of the study team observes significant distress during their interaction with a survivor, a referral to a psychologist, psychiatrist, or other relevant healthcare professional will be offered. The survivor’s intervention can continue or be aborted depending on the response of the participant. If any disclosures of risks to safety (e.g., suicidal ideation) occur during any stages of the study, standard clinical processes will be followed including safety planning with the participant, and when needed advising an appropriate support person such as a member of the participant’s treating team and/or a family member. This limit to confidentiality is included in the consent form.

The sponsor and ethics department will be notified immediately of any safety issues and the management of these, respectively.

### Evaluation of Fear-Less by clinicians

#### Participants

Clinical staff working in the breast, gynecology, and head and neck clinical services will be invited to evaluate the *Fear-Less* program. These clinical staff may include nurses, oncologists, psychosocial oncology staff, and allied health staff.

##### Sample size

All clinical staff involved in the *Fear-Less* program will be invited to participate. It is anticipated that ten staff will be invited.

##### Recruitment and consent

Staff will be invited to provide feedback regarding their experiences with the program via an online survey. Staff will be emailed a link to the survey towards the end of the *Fear-Less* program evaluation period. It is anticipated this will occur in the last 1 to 2 weeks of the recruitment period and continue until the final participant has completed their intervention. Consent is implied by completion and return of the questionnaire.

#### Study measures

##### Staff evaluation survey

The extent to which *Fear-Less* is acceptable for clinicians will be assessed using the *Staff Evaluation Survey* (see Additional File 2), a purpose developed 10-item measure assessing each staff member’s involvement in the program and includes open-response items requesting positive and negative feedback. This survey was adapted from our earlier study evaluating *Fear-Less* among survivors with stage IV melanoma [32].

##### Process data

To further assess feasibility, the CRF will be used by the project team to record the time taken (in minutes) to deliver each component of the intervention and follow-up care (e.g., follow-up phone calls and referrals following intervention).

### Data management

Data will be entered and managed through REDCap [40, 41] and stored securely in password-protected folders on secure servers at Peter Mac. Only members of the research team will have access to this data, in accordance with the National Statement on Ethical Conduct in Human Research 2018 and the Australian Code for Responsible Conduct of Research 2018. Hard-copy data will be stored in locked filing cabinets within the Department of Psychosocial Oncology at Peter Mac. Five years after publication or dissemination of project outcomes, hard-copy and electronic data will be destroyed.

Anonymous data from the FCRI-SF and FCR-1 may also be shared with each measure’s author to assist in their ongoing validation of these measures. The data shared may also include anonymous demographic and medical information to describe the population from which the data is drawn. This data will be anonymized in a separate password-protected file; the password will be provided to the author separately to the emailed data file.

### Data analysis

#### Quantitative analysis

Quantitative data will be analyzed using SPSS (V.24).

##### Descriptive statistics

Descriptive statistics (e.g., count/percentage, mean/SD, median, interquartile range) will be used to summarise demographic, clinical and process data, responses to the FCRI-SF and FCR-1, and evaluation survey questions.

##### Feasibility

The feasibility of the stepped-care approach will be assessed by calculating percentages of survivors who completed screening, percentage of participants who consented to the interventions, percentage adherence to the interventions, and clinician time to deliver screening and interventions. The feasibility criteria (Table 2) are based on previous studies [11, 32, 42].

**Table 2:** Feasibility criteria for *Fear-Less*

Outcome	Feasibility criteria
Screening rate	A majority (>60%) of survivors completing screening
Intervention uptake	A majority (>60%) of participants consenting to the intervention
Intervention adherence	• For those receiving the self-management resource, self-reported reading 75% of more of the booklet by two thirds of participants • For those receiving ConquerFear, completion of four or more sessions of the intervention

##### Impact

Change scores between pre- and post-intervention FCRI-SF will be calculated to describe the impact of the *Fear-Less* interventions. Participants who report a reduction of 10% or more on the FCRI-SF (indicative of a clinically significant improvement [43]) will be considered to have a reduction in FCR.

##### Exploratory analysis of FCR-1

Criterion validity [44] of the FCR-1 will be assessed using linear regression to examine associations between FCR-1 and FCRI-SF scores while controlling for known correlates of FCR, such as age and gender. Coefficients exceeding *r*=0.7 will be considered satisfactory [45].

A receiver operator characteristic (ROC) analysis will evaluate the ability of the FCR-1 to differentiate between clinical and non-clinical levels of FCR, as indicated by the classification criteria of a FCRI-SF score ≥22/36. An optimal cut-off score will also be determined.

#### Qualitative data analysis

To assess acceptability, free text items from the survivor and clinician experience surveys will be analyzed using content analysis. A deductive approach will be used for coding data. Pre-defined categories will be formulated based on the research questions informing the study. Additional inductive codes will be identified from the survey responses.

### Dissemination

The outcomes of this study may be published or presented at seminars and conferences. No identifying information will be made available during the publications or presentations. The resources developed as part of this study may also be made publicly available to other hospitals and organizations. This aims to broaden the reach and accessibility of resources for cancer survivors experiencing FCR.

## Discussion

Approximately 50% of cancer survivors experience moderate-to-high levels of FCR [2, 3], which left untreated, are associated with poorer health outcomes [5, 7–9]. Support for FCR remains a significant unmet need for cancer survivors, where systemic barriers to identifying and treating FCR include lack of routine screening and limited resources to provide interventions for individuals experiencing FCR [22, 23]. Specific strategies, such as a stepped-care approach and the refinement of interventions included in this approach, may provide a solution to these barriers [26].

The *Fear-Less s*tepped-care model provides a framework for screening FCR and stratifying survivors to the appropriate level of treatment, so that the more intensive ConquerFear is reserved for individuals with clinical FCR. This is a novel model of care that has shown promising outcomes with a stage IV melanoma population [32]. Our study will provide an indication of whether this model is also feasible and acceptable amongst early-stage cancer survivors.

Additionally, our study will contribute to the growing literature regarding less intensive FCR interventions. As described by Pradhan et al. [26], there are currently few minimal interventions for FCR that can be implemented within a stepped-care approach. The outcomes from our evaluation will provide an indication of whether our purpose-developed self-management resource is acceptable and feasible to participants and improves FCR levels.

Should this be the case, further investigations regarding the efficacy and cost-effectiveness of *Fear-Less* can be conducted.

## Supplementary Information

**Additional File 1.** The SPIRIT Checklist.

**Additional File 2.** Copies of the 3 Survivor Experience Surveys and 1 Staff Engagement Survey referenced in the manuscript.

## Data Availability

Not applicable as no datasets were generated or analyzed during this study.

## Abbreviations

FCR: Fear of cancer recurrence
FCRI-SF: Fear of Cancer Recurrence Inventory—Short Form
FCR-1: Fear of Cancer Recurrence—One Item Measure
PICF: Participant information and consent form
CRF: Case-report form

## References

[1] Lebel S, Ozakinci G, Humphris G, Mutsaers B, Thewes B, Prins J (2016). From normal response to clinical problem: definition and clinical features of fear of cancer recurrence. Support Care Cancer..

[2] Smith AB, Costa D, Galica J, Lebel S, Tauber N, van Helmondt SJ (2020). Spotlight on the Fear of Cancer Recurrence Inventory (FCRI). Psychol Res Behav Manage.

[3] Casswell G, Gough K, Drosdowsky A, Bressel M, Coleman A, Shrestha S (2021). Fear of cancer recurrence in survivors of human papillomavirus−associated oropharyngeal carcinoma. Int J Radiation Oncol Biol Phys.

[4] Mutsaers B, Butow P, Dinkel A, Humphris G, Maheu C, Ozakinci G (2020). Identifying the key characteristics of clinical fear of cancer recurrence: an international delphi study. Psycho Oncol.

[5] Hart SL, Latini DM, Cowan PR, Carroll JE, Investigators C (2008). Fear of recurrence, treatment satisfaction, and quality of life after radical prostatectomy for prostate cancer. Support Care Cancer..

[6] Kasparian NA, McLoone JK, Butow PN (2009). Psychological responses and coping strategies among patients with malignant melanoma: a systematic review of the literature. Arch Dematol.

[7] Maheu C, Lebel S, Courbasson C, Lefebvre M, Singh M, Bernstein LJ (2016). Protocol of a randomized controlled trial of the fear of recurrence therapy (FORT) intervention for women with breast or gynecological cancer. BMC Cancer..

[8] Glynne-Jones R, Chait I, Thomas SF (1997). When and how to discharge cancer survivors in long term remission from follow-up: the effectiveness of a contract. Clin Oncol.

[9] Williams JTW, Pearce A, Smith AB (2021). A systematic review of fear of cancer recurrence related healthcare use and intervention cost-effectiveness. Psycho Oncol.

[10] Butow P, Turner J, Gilchrist J, Sharpe L, Smith AB, Fardell JE (2017). Randomized trial of conquerfear: a novel, theoretically based psychosocial intervention for fear of cancer recurrence. J Clin Oncol.

[11] Dieng M, Butow PN, Costa DSJ, Morton RL, Menzies SW, Mireskandari S, et al. Psychoeducational intervention to reduce fear of cancer recurrence in people at high risk of developing another primary melanoma: results of a randomized controlled trial. J Clin Oncol. 2016;34(36):4405–14. 10.1200/JCO.2016.68.2278 27998215

[12] Herschbach P, Book K, Dinkel A, Berg P, Waadt S, Duran G (2010). Evaluation of two group therapies to reduce fear of progression in cancer patients. Support Care Cancer.

[13] van de Wal M, Thewes B, Gielissen M, Speckens A, Prins J (2017). Efficacy of blended cognitive behavior therapy for high fear of recurrence in breast, prostate, and colorectal cancer survivors: the SWORD study, a randomized controlled trial. J Clin Oncol.

[14] Humphris GM, Rogers SN (2012). AFTER and beyond: cancer recurrence fears and a test of an intervention in oral and oropharyngeal patients. Soc Sci Dentistry..

[15] Lichtenthal WG, Corner GW, Slivjak ET, Roberts KE, Li Y, Breitbart W (2017). Effects of a randomized gratitude intervention on death-related fear of recurrence in breast cancer survivors. Cancer..

[16] Otto AK, Szczesny EC, Soriano EC, Laurenceau J, Siegel SD (2016). Effects of a randomized gratitude intervention on death-related fear of recurrence in breast cancer survivors. Health Psychol.

[17] Tauber NM, O'Toole MS, Dinkel A, Galica J, Humphris G, Lebel S (2019). Effect of psychological intervention on fear of cancer recurrence: a systematic review and meta-analysis. J Clin Oncol.

[18] Fardell JE, Thewes B, Turner J, Gilchrist J, Sharpe L, Smith AB (2016). Fear of cancer recurrence: a theoretical review and novel cognitive processing formulation. J Cancer Survivor.

[19] Sharpe L, Turner J, Fardell JE, Thewes B, Smith AB, Gilchrist J (2019). Psychological intervention (ConquerFear) for treating fear of cancer recurrence: mediators and moderators of treatment efficacy. J Cancer Survivor.

[20] Butow LP, Williams D, Thewes B, Tesson S, Sharpe L, Smith AB (2018). A psychological intervention (ConquerFear) for treating fear of cancer recurrence: views of study therapists regarding sustainability. Psycho Oncol.

[21] Shih STF, Butow P, Bowe SJ, Thewes B, Turner J, Gilchrist J, et al. Cost-effectiveness of an intervention to reduce fear of cancer recurrence: the ConquerFear randomized controlled trial. Psycho Oncol. 2019;28:1071–9. 10.1002/pon.5056 30860653

[22] Lisy K, Langdon L, Piper A, Jefford M (2019). Identifying the most prevalent unmet needs of cancer survivors in Australia: a systematic review. Asia Pacific J Clin Oncol.

[23] Smith AB (2021). Integrating fear of cancer recurrence screening into routine care: opportunities and challenges. Psycho Oncol.

[24] Butow P, Shaw J, Vaccaro L, Sharpe L, Dhillon H, Smith B (2019). A research agenda for fear of cancer recurrence: a delphi study conducted in Australia. Psycho Oncol.

[25] Bower P, Gilbody S (2005). Stepped care in psychological therapies: access, effectiveness and efficiency. Bri J Psychiatry..

[26] Pradhan P, Sharpe L, Menzies RE (2021). Towards a stepped care model for managing fear of cancer recurrence or progression in cancer survivors. Cancer Manage Res.

[27] Jansen F, Krebber AMH, VMH C’e, Cuijpers P, de Bree R, Becker-Commissaris A (2017). Cost-utility of stepped care targeting psychological distress in patients with head and neck or lung cancer. J Clin Oncol.

[28] Singer S, Danker H, Roick J, Einenkel J, Briest S, Spieker H (2017). Effects of stepped psychooncological care on referral to psychosocial services and emotional well-being in cancer patients: a cluster-randomized phase III trial. Psycho Oncol.

[29] Zhou ES, Michaud AL, Recklitis CJ (2020). Developing efficient and effective behavioral treatment for insomnia in cancer survivors: results of a stepped care trial. Cancer..

[30] Jansen F, Lissenberg-Witte B, Krebber A, Cuijpers P, Bree R, Becker-Commissaris A (2019). Stepped care targeting psychological distress in head and neck cancer and lung cancer patients: which groups specifically benefit? Secondary analyses of a randomized controlled trial. Support Care Cancer..

[31] Cancer Australia. Recommendations for the identification and management of fear of cancer recurrence in adult cancer survivors. Cancer Australia, Sydney. 2013

[32] Lynch FA, Katona K, Jefford M, Smith AB, Shaw J, Dhillon HM (2020). Feasibility and acceptability of fear-less: a stepped-care program to manage fear of cancer recurrence in people with metastatic melanoma. J Clin Med.

[33] Lewis M, Bromley K, Sutton CJ, McCray G, Myers HL, Lancaster GA. Determining sample size for progression criteria for pragmatic pilot RCTs: the hypothesis test strikes back! Pilot and Feasibility Studies. 2021;7:40. 10.1186/s40814-021-00770-x PMC7856754 33536076

[34] Billingham SAM, Whitehead AL, Julious SA. An audit of sample sizes for pilot and feasibility trials being undertaken in the United Kingdom registered in the United Kingdom Clinical Research Network database. BMC Med Res Methodol. 2013;13:104. 10.1186/1471-2288-13-104 PMC3765378 23961782

[35] Simard S, Savard J (2009). Fear of Cancer Recurrence Inventory: development and initial validation of a multidimensional measure of fear of cancer recurrence. Support Care Cancer..

[36] Rudy L, Maheu C, Körner A, Lebel S, Gélinas C (2020). The FCR-1: initial validation of a single-item measure of fearof cancer recurrence. Psycho Oncol.

[37] Proctor E, Silmere H, Raghavan R, Hovmand P, Aarons G, Bunger A (2011). Outcomes for implementation research: conceptual distinctions, measurement challenges, and research agenda. Admin Policy Mental Health Mental Health Serv Res.

[38] Fardell JE, Jones G, Smith AB, Lebel S, Thewes B, Costa D (2018). Exploring the screening capacity of the Fear of Cancer Recurrence Inventory-Short Form for clinical levels of fear of cancer recurrence. Psycho Oncol.

[39] Simard S, Savard J (2015). Screening and comorbidity of clinical levels of fear of cancer recurrence. J Cancer Survivor.

[40] Harris PA, Taylor R, Minor BL, Elliott V, Fernandez M, O’Neal L, et al. The REDCap consortium: building an international community of software partners. J Biomed Inform. 2019;95:103208. 10.1016/j.jbi.2019.103208 PMC7254481 31078660

[41] Harris PA, Taylor R, Thielke R, Payne J, Gonzalez N, Conde JG (2009). A metadata-driven methodology and workflow process for providing translational research informatics support. J Biomed Inform.

[42] Tauber NM, Zachariae R, Jensen AB, Thewes B, Skyt I, Elkjær E (2022). ConquerFear-group: Feasibility study with pilot results of a psychological intervention for fear of cancer recurrence delivered in groups. Psycho Oncol.

[43] Brown LD, Cai TT, Dasgupta A (2001). Interval estimation for a binomial proportion. Stat Sci.

[44] Boateng GO, Neilands TB, Frongillo EA, Melgar-Quiñonez HR, Young SL (2018). Best practices for developing and validating scales for health, social, and behavioral research: a primer. Front. Public Health.

[45] Terwee CB, Bot SDM, de Boer MR, van der Windt DAWM, Knol DL, Dekker J (2007). Quality criteria were proposed for measurement properties of health status questionnaires. J Clin Epidemiol.

