# Supplementary material for: Study protocol for the evaluation of Fear-Less: a stepped-care program for fear of cancer recurrence in survivors with early-stage disease
Source: Pilot Feasibility Stud. 2022 Aug 10;8:177. doi: 10.1186/s40814-022-01123-y (PMC9364569; doi:10.1186/s40814-022-01123-y)
Supplement: Supplementary file 2 — Additional File 2. Copies of the 3 Survivor Experience Surveys and 1 Staff Engagement Survey referenced in the manuscript. [file 40814_2022_1123_MOESM2_ESM.docx]

**Survivor Experience Survey**

**Screening Questionnaires**

As part of your routine follow-up appointments with your medical team, you have completed two short screening questionnaires asking about your level of fear of cancer returning. This survey focuses on your experiences completing these questionnaires. The survey may take up to 10 minutes of your time.

Thank you for agreeing to participate in this survey.

Your responses are confidential, and you don’t need to answer any questions if you don’t want to.

| **Date:** _______/_________/_________ | | | | | | | |
| --- | --- | --- | --- | --- | --- | --- | --- |
| 1. **Prior to this program, has anyone ever discussed fear of cancer returning with you?** | | | | | | | |
| _0_ No | | | | | | | |
| _1_ Yes | | | | | | | |
| If yes, please tell us who discussed this with you and what happened next?  ________________________________________________________________________________  ________________________________________________________________________________ | | | | | | | |
| 1. **Prior to this program, have you previously received information, treatment or support to improve your fear of cancer returning?** | | | | | | | |
| _0_ No (proceed to Question 4) | | | | | | | |
| _1_ Yes (please complete Q2a and 2b)  **2a. Please specify the type of support you received for your fear:**  _0_  Psychological treatment  _1_  Medical treatment  _2_  Professional advice. Explain ____________________________________  _3_  Information or fact sheets  _4_ Other: ______________________________________________________  **2b. Please specify who recommended this support**  _0_  I sought it myself  _1_  My medical team (e.g., oncologist; doctors; nurses)  _2_  My friends or family  _3_  Other: ______________________________________________________ | | | | | | | |
| 1. **What has stopped you from seeking help for your fear of cancer returning in the past?** | | | | | | | |
| _0_  I did not have the time  _1_  I was embarrassed about seeking help  _2_  I did not know what to expect from the help or support  _3_  I was worried I would feel more upset or anxious  _4_ I felt that I had enough support from elsewhere  _5_ I thought that I did not need help  _6_ Other: _______________________________________________________ | | | | | | | |
| 1. **Thinking about the screening questionnaires you completed, what format did you use to complete the questionnaires?**   _1_  In person _2_  Phone _3_ Online (via email or link) _4_ Returned by mail | | | | | | | |
| 1. **Were the reasons & procedures for completing the screening questionnaires well-explained?** | | | | | | | |
| _1_ No | | _2_ Yes |  | |  |  | |
| 1. **The screening questionnaires procedures were easy to complete.** | | | | | | | |
| _1_  **Strongly disagree** | | _2_  **Disagree** | _3_  **Don’t know** | | _4_  **Agree** | _5_  **Strongly Agree** | |
| 1. **The screening questionnaires procedures were easy to understand.** | | | | | | | |
| _1_  **Strongly disagree** | | _2_  **Disagree** | _3_  **Don’t know** | | _4_  **Agree** | _5_  **Strongly Agree** | |
| 1. **The time it took to complete the screening questionnaires was acceptable.** | | | | | | | |
| _1_  **Strongly disagree** | | _2_  **Disagree** | _3_  **Don’t know** | | _4_  **Agree** | _5_  **Strongly Agree** | |
| 1. **Which questionnaire best described your experience?** | | | | | | | |
|  | _1_ **Fear of Cancer Recurrence Inventory – Short Form** **(FCRI-SF)**  (first questionnaire) | |  | _2_ **Fear of Cancer Recurrence – 1 Item Measure (FCR-1)**  (second questionnaire) | | |  |
| 1. **How important do you think it is to screen for fear of cancer returning?**   **When do you think is the best time to screen for fear of cancer returning?**  **_____________________________________________________________________________________________**  **_____________________________________________________________________________________________** | | | | | | | |

**Please add any additional comments or feedback about the screening questionnaires.**

**________________________________________________________________________________________________**

**________________________________________________________________________________________________**

**________________________________________________________________________________________________**

**________________________________________________________________________________________________**

**Thank you for participating in this survey.**

**Survivor Experience Survey**

**Post Follow-Up Care: Self-Management Intervention**

Thank you for agreeing to participate in this survey about the ‘Fear-Less’ Program. You have been asked to complete this survey because you received the self-management intervention. When you see the term ‘self-management intervention’ we mean the booklet and the follow up phone calls you received.

This survey is about your experiences with the self-management intervention. The survey may take up to 10 minutes of your time. Your responses are confidential, and you are free to withdraw from the survey at any stage.

| **Date:** _______/_________/____________ | | | | |
| --- | --- | --- | --- | --- |
| 1. **Thinking about the ‘Fear-Less’ Program self-management intervention you have just received, please rate the amount of the booklet that you have read:** | | | | |
| ☐_0_ 0% of the booklet (none) | ☐_1_  25% of the booklet | ☐_2_ 50% of the booklet | ☐_3_  75% of the booklet | ☐_4_ 100% of the booklet |
| 1. **How many exercises in the booklet did you complete?** (i.e., Getting To Know Your Triggers; Relaxation Diary; Thought Diary; Looking At Your Control; Finding Your Values; Setting Goals; Your Plan To Manage Fear) | | | | |

| ☐_0_ 0 | ☐_1_  1 | ☐_2_ 2 | | ☐_3_ 3 | ☐_4_  4 | ☐_5_ 5 | | ☐_6_ 6 | ☐_7_ 7 |
| --- | --- | --- | --- | --- | --- | --- | --- | --- | --- |
| 1. **Which of the exercises in the booklet were most helpful?** (You can tick more than one)   _1_  Getting To Know Your Triggers  _2_  Relaxation Diary  _3_  Thought Diary  _4_  Looking At Your Control  _5_ Finding Your Values  _6_ Setting Goals  _7_ Your Plan To Manage Fear  _8_ Other (please specify) _________________________________________________________ | | | | | | | | | |
| 1. **Thinking about the ‘Fear-Less’ Program self-management intervention you have just received, what aspects of your care were most useful and helpful?** | | | | | | | | | |
| _________________________________________________________________________________  _________________________________________________________________________________  _________________________________________________________________________________ | | | | | | | | | |
| 1. **Thinking about the ‘Fear-Less’ Program self-management intervention you have just received, what aspects of your care were least useful and helpful?** | | | | | | | | | |
| _________________________________________________________________________________  _________________________________________________________________________________  _________________________________________________________________________________ | | | | | | | | | |
| 1. **Which of the following best describes any change in your fear of cancer returning since receiving the ‘Fear-Less’ self-management intervention?** (please tick) | | | | | | | | | |
| _1_ | My fear is much worse | | | | | | | | |
| _2_ | My fear is somewhat worse | | | | | | | | |
| _3_ | My fear has not changed | | | | | | | | |
| _4_ | My fear is somewhat better | | | | | | | | |
| _5_ | My fear is much better | | | | | | | | |
| 1. **To what extent do you think the changes in your fear are related to your self-management intervention?** | | | | | | | | | |
| _1_  **Not at all related** | | | _2_  **Partly related** | | | | _3_  **Completely related** | | |
| 1. **What is your preferred way of receiving the Fear-Less follow-up care for fear of cancer returning?** (Please tick) | | | | | | | | | |
| _1_ | Telephone | | | | | | | | |
| _2_ | Video-conferencing | | | | | | | | |
| _3_ | Face-to-face by myself (one on one with a clinician) | | | | | | | | |
| _4_ | Face-to face in a group | | | | | | | | |
| _5_ | Online | | | | | | | | |
| _6_ | Paper based resource/information | | | | | | | | |
| 1. **Would you recommend the Fear-Less program to other patients with cancer?** | | | | | | | | | |
| _1_  **Yes** | | | _2_  **Don’t know** | | | | _3_  **No** | | |
| 1. **What are your suggestions about how we can improve the ‘Fear-Less’ Program or the intervention you received?** | | | | | | | | | |
| _________________________________________________________________________________  _________________________________________________________________________________  _________________________________________________________________________________ | | | | | | | | | |

**Thank you for participating in the survey.**

**Survivor Experience Survey**

**Post Follow-Up Care: ConquerFear Treatment**

Thank you for agreeing to participate in this survey about the ‘Fear-Less’ Program. You have been asked to complete this survey because you received a referral for the ConquerFear treatment and have completed that follow-up care.

This survey is about your experiences with the ConquerFear treatment. The survey may take up to 10 minutes of your time. Your responses are confidential, and you are free to withdraw from the survey at any stage.

| **Date:** _______/_________/____________ | | |
| --- | --- | --- |
| 1. **Thinking about the ‘Fear-Less’ Program ConquerFear treatment you have just received, what aspects of your care were most useful and helpful?** | | |
|  | __________________________________________________________________________________  __________________________________________________________________________________  __________________________________________________________________________________ | |
| 1. **Thinking about the ‘Fear-Less’ Program ConquerFear treatment you have just received, what aspects of your care were least useful and helpful?** | | |
|  | __________________________________________________________________________________ | |
|  | __________________________________________________________________________________  __________________________________________________________________________________ | |
| 1. **Which of the following best describes any change in your fear of cancer returning since receiving your ConquerFear treatment in the ‘Fear-Less’ Program?** (please tick) | | |
| _1_ | | My fear is much worse |
| _2_ | | My fear is somewhat worse |
| _3_ | | My fear has not changed |
| _4_ | | My fear is somewhat better |
| _5_ | | My fear is much better |

| 1. **To what extent do you think the changes in your fear are related to your ConquerFear treatment?** | | | | | |
| --- | --- | --- | --- | --- | --- |
| _1_  **Not at all related** | | | | _2_  **Partly related** | _3_  **Completely related** |
| 1. **What is your preferred way of receiving the ConquerFear treatment for fear of cancer returning?** (Please tick) | | | | | |
| _1_ | | Telephone | | | |
| _2_ | | Video-conferencing | | | |
| _3_ | | Face-to-face by myself (one on one with a clinician) | | | |
| _4_ | | Face-to face in a group | | | |
| _5_ | | Online | | | |
| _6_ | | Paper based resource/information | | | |
| 1. **If you received the ConquerFear treatment via telehealth, what did you like about these appointments?** (Please tick) | | | | | |
| _1_ | | It was more convenient to attend appointments from home | | | |
| _2_ | | I felt more comfortable in my own home (e.g., I felt I had more privacy) | | | |
| _3_ | | I had no trouble logging on to the telehealth platform | | | |
| _4_ | | I felt connected with my psychologist | | | |
| _5_ | | I found it easy to practice the exercises via telehealth | | | |
| _6_ | | Other (please specify):  __________________________________________________________________________________  __________________________________________________________________________________ | | | |
| 1. **If you received the ConquerFear treatment via telehealth, what did you dislike about these appointments?** (Please tick) | | | | | |
| _1_ | | I had internet connection issues | | | |
| _2_ | | I felt less comfortable in my own home (e.g., I felt I had less privacy) | | | |
| _3_ | | I had trouble logging on to the telehealth platform | | | |
| _4_ | | I felt disconnected from my psychologist | | | |
| _5_ | | I found it hard to practice the exercises via telehealth | | | |
| _6_ | | Other (please specify):  __________________________________________________________________________________  __________________________________________________________________________________ | | | |
| 1. **Would you recommend the Fear-Less program to other patients with cancer?** | | | | | |
| _1_  **Yes** | | | _2_  **Don’t know** | | _3_  **No** |
| 1. **What are your suggestions about how we can improve the ‘Fear-Less’ Program or the psychology treatment you received?** | | | | | |
|  | __________________________________________________________________________________  __________________________________________________________________________________ __________________________________________________________________________________ __________________________________________________________________________________ | | | | |

**Thank you for participating in the survey.**

**Staff Engagement Survey**

**Statement of Participation**

**Please read the following information before completing this survey.**

Fear of cancer recurrence is a common complaint of cancer patients. To help survivors of early stage cancers with their fear of recurrence, we have implemented a stepped-care program across Peter Mac, Royal Melbourne Hospital, and Royal Women’s Hospital called the Fear-Less Program. The Fear-Less Program screens and identifies patients with fear of cancer recurrence, and provides those patients with an appropriate referral to either self-management intervention or individual therapy through the psychology department at their treating hospital.

You have been invited to participate in this survey because you are health professional working with cancer patients and have been involved in the Fear-Less Program. Your involvement may have been direct, that is you have delivered a key intervention, or it may be that you were involved in the screening and referral of patients to this program.

We are interested in your experiences with the Fear-Less Program. Your participation in this survey is voluntary and anonymous. Your responses to this survey will be stored securely at Peter Mac for 7 years. When the results of this survey are presented or published, no details which may identify you will be reported.

This project has been approved by the Peter Mac Human Research Ethics Committee. The Fear-Less Program and evaluation project has been funded by the Victorian Cancer Survivorship Program: Phase II Grants Scheme. If you have any questions or concerns about this survey, or would like more information about the study, please contact Dr Maria Ftanou, on 85595220 or [Maria.Ftanou@petermac.org](mailto:Maria.Ftanou@petermac.org). If you wish to contact the Peter Mac Ethics Committee, please call 855 97540 or email [ethics@petermac.org](mailto:ethics@petermac.org)

This survey consists of 10 questions and should take about 20 minutes to complete. We are interested in your feedback on what we can do to improve the Fear-Less Program.

Thank you for your participation – we are grateful for your time and your feedback.

| 1. **What is your profession?** |  |
| --- | --- |
| 1. **What is your role in the service?** |  |
| 1. **How long have you been working in healthcare?** |  |
| 1. **What assistance did you provide patients with fear of cancer recurrence prior to the Fear-Less Program?** | Medication prescription  Referrals (e.g., to GP; psychologists)  Delivering psychological interventions  Practical support / advice  None / Not Applicable |
| 1. **What was your involvement with the Fear-Less Program?** | Patient identification  Patient screening  Advising patients of the project  Referrals to Fear-Less project team  Delivering follow-up care (self-management or individual therapy)  Advisory / project team  No direct involvement  Other (please specify)……………………………………. |
| 1. **Based on your experiences, how would you improve the Fear-Less Program?** |  |
| 1. **What impact (positive and negative) do you believe the Fear-Less Program has had on patients and patient care?** |  |
| 1. **Overall, what impact (positive and negative) do you believe the Fear-Less Program has had on your service?** |  |
| 1. **What further information would you like on the Fear-Less Program or information and training on fear of cancer recurrence in general?** |  |
| 1. **Do you have any additional comments about the Fear-Less Program?** |  |

**Thank you for participating.**
